# Delta‐9‐tetrahydrocannabinol disrupts mitochondrial function and attenuates syncytialization in human placental BeWo cells

**DOI:** 10.14814/phy2.14476

**Published:** 2020-07-06

**Authors:** O’Llenecia S. Walker, Rehginald Ragos, Harmeet Gurm, Mariah Lapierre, Linda L. May, Sandeep Raha

**Affiliations:** ^1^ Department of Pediatrics McMaster University Hamilton ON Canada; ^2^ The Graduate Program in Medical Sciences McMaster University Hamilton ON Canada

**Keywords:** cannabinoid, mitochondria, oxidative stress, placenta, trophoblasts

## Abstract

The psychoactive component in cannabis, delta‐9‐tetrahydrocannabinol, can restrict fetal growth and development. Delta‐9‐tetrahydrocannabinol has been shown to negatively impact cellular proliferation and target organelles like the mitochondria resulting in reduced cellular respiration. In the placenta, mitochondrial dysfunction leading to oxidative stress prevents proper placental development and function. A key element of placental development is the proliferation and fusion of cytotrophoblasts to form the syncytium that comprises the materno‐fetal interface. The impact of delta‐9‐tetrahydrocannabinol on this process is not well understood. To elucidate the nature of the mitochondrial dysfunction and its consequences on trophoblast fusion, we treated undifferentiated and differentiated BeWo human trophoblast cells, with 20 µM delta‐9‐tetrahydrocannabinol for 48 hr. At this concentration, delta‐9‐tetrahydrocannabinol on BeWo cells reduced the expression of markers involved in syncytialization and mitochondrial dynamics, but had no effect on cell viability. Delta‐9‐tetrahydrocannabinol significantly attenuated the process of syncytialization and induced oxidative stress responses in BeWo cells. Importantly, delta‐9‐tetrahydrocannabinol also caused a reduction in the secretion of human chorionic gonadotropin and the production of human placental lactogen and insulin growth factor 2, three hormones known to be important in facilitating fetal growth. Furthermore, we also demonstrate that delta‐9‐tetrahydrocannabinol attenuated mitochondrial respiration, depleted adenosine triphosphate, and reduced mitochondrial membrane potential. These changes were also associated with an increase in cellular reactive oxygen species, and the expression of stress responsive chaperones, *HSP60* and *HSP70*. These findings have important implications for understanding the role of delta‐9‐tetrahydrocannabinol‐induced mitochondrial injury and the role this might play in compromising human pregnancies.

## Graphical abstract

THC disrupted mitochondrial function, increased markers of mitochondrial fission and cellular stress in BeWo cells. This was coincident with reduced BeWo cell fusion and secretion of important fetal growth signals, hPL and IGF2. These changes were mediated, in part, via the CB1 receptor in syncytialized BeWo cells.

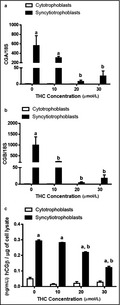

## 1 INTRODUCTION

Cannabis is a commonly used recreational drug among pregnant women (Alpár, Di Marzo, & Harkany, 2016; Wu, Jew, & Lu, 2011), and its legalization in many states as well as Canada may lead to the perception that it does not contribute to adverse pregnancy outcomes and is safe to use. Data collected from 2002 to 2014 in the US show that 7.5% of pregnant women between 18 and 25 years of age smoke or otherwise consume cannabis, while the rate of use across all pregnant women is approximately 4% (Brown et al., 2017). The therapeutic benefits of cannabis use, including its antiemetic and analgesic effects (Chakravarti, Ravi, & Ganju, 2014; Parker, Rock, & Limebeer, 2014; Webb & Webb, 2014), make it an attractive drug of choice for pregnant women to manage the related symptoms of pregnancy, particularly because these women may view cannabis as natural product. Although there are presently no reported teratogenic effects in rodent or human studies, cannabis use has been implicated in neurodevelopmental disorders in the offspring (Grant, Petroff, Isoherranen, Stella, & Burbacher, 2018; de Salas‐Quiroga et al., 2015; Vela et al., 1998). The main psychoactive constituent, delta‐9‐tetrahydrocannabinol (THC), is lipophilic and it has been reported that approximately one third of THC in the maternal circulation can cross the placenta (Wu et al., 2011) and/or (a) directly act on its molecular targets in the fetus, (b) act within the decidua/placenta, with the potential to attenuate placental secretions/signaling to the fetus or uteroplacental blood flow, and lastly (c) exert negative effects on maternal physiology which may, by secondary means, influence the fetus, such as increased secretion of stress‐related hormones (Ross, Graham, Money, & Stanwood, 2015). Cannabinoids, including THC, largely exert their biological effects via the activation of G protein‐coupled cannabinoid receptors—CB1 and CB2; an integral part of the endocannabinoid system (Bénard et al., 2012; Costa, 2016a; Habayeb, Taylor, Bell, Taylor, & Konje, 2008). CB1 and CB2 are expressed in several organs and tissues and participate in multiple physiological events (Howlett et al., 2002). Both of these receptors have also been identified on term placentae and in the BeWo choriocarcinoma cell line (Costa, 2016a). Cannabinoid receptors have also been localized on the mitochondria (Bénard et al., 2012), which are found in abundance in placentae (Casanueva & Viteri, 2003), a highly metabolically active organ (Mandò et al., 2014). While mitochondrial dysfunction is known to be linked to poor placental development (Maloyan, Mele, Muralimanohara, & Myatt, 2012; Mandò et al., 2014; Poidatz, Dos Santos, Gronier, et al., 2015), whether such associations exist for cannabis use during pregnancy is not well defined. Restricted fetal growth, the most common finding that is associated with in utero cannabis exposure (Costa, 2016a), may be associated with placental pathology stemming from impaired trophoblast function. We, along with others, have recently demonstrated that compromised placental development can be correlated with reduced fetal growth (Benevenuto et al., 2017; Deyssenroth et al., 2017; Hayes et al., 2012; Natale et al., 2020).

Two lineages of trophoblasts are primarily involved in the formation of the materno‐fetal interface: cytotrophoblasts and syncytiotrophoblasts. Cytotrophoblasts exist in close proximity to the syncytiotrophoblasts and can fuse to give rise to multinucleated syncytium which forms the materno‐fetal interface. The syncytium is found in direct contact with maternal blood, thus it mediates the exchange of oxygen, nutrients, gasses, and waste products, and is primarily responsible for the endocrine functions of the placenta, secreting hormones such as human chorionic gonadotropin (hCG), human placental lactogen (hPL), and progesterone (Gude, Roberts, Kalionis, & King, 2004). The biochemical and morphological differentiation of cytotrophoblasts into syncytiotrophoblasts is critical for proper placental development, and consequently, for a healthy pregnancy (Gupta, Malhotra, Malik, Verma, & Chaudhary, 2015). For example, THC has been shown to attenuate glucose (Araújo, Gonçalves, & Martel, 2008) and folic acid (Keating, Gonçalves, Campos, Costa, & Martel, 2009) uptake by the syncytial barrier as well as inhibit the proliferation of BeWo cells (Khare, Taylor, Konje, & Bell, 2006) and impair the differentiation from cytotrophoblasts to syncytiotrophoblasts (Costa, Fonseca, Marques, Teixeira, & Correia‐da‐Silva, 2015) in human trophoblasts. In vivo, treatment of mice with THC during pregnancy resulted in reduced number of pups and lower maternal and placental weights (Chang et al., 2017).

While it has been demonstrated that THC impairs mitochondrial function in neuronal systems (Athanasiou et al., 2007; Wolff et al., 2015), the consequences of THC on trophoblast mitochondrial dynamics, respiration, and associated stress responses have not been fully explored. Given that mitochondrial dysfunction, oxidative stress, and poor trophoblast outcomes (Walker et al., 2020), and THC exposure during pregnancy leading to poor placentation and restricted intrauterine growth (Natale et al., 2020) has been demonstrated in rats, we hypothesize that THC may directly increase oxidative stress and reduce ATP generation, and alter trophoblast gene expression resulting in attenuated syncytialization. We report that exposure of human‐derived BeWo cells to concentrations of THC found in frequent users (Cherlet & Scott, 2002; Khare et al., 2006) results in reduced mitochondrial membrane potential, reduced mitochondrial respiration, and increased cellular reactive oxygen production. Functionally, these changes were concomitant with reduced BeWo cell syncytialization and transcription of important fetal growth hormones.

## 2 MATERIALS AND METHODS

### 2.1 Cell culture

All cell culture experiments were carried out under McMaster University Biohazard Utilization Protocol BUP023. BeWo cells (ATCC^®^ CCL‐98) were routinely grown and maintained in Hams F‐12K medium supplemented with 10% FBS, 1% penicillin/streptomycin, and 1% l‐glutamine, maintained in a humidified atmosphere of 5% CO_2_ at 37°C. Undifferentiated BeWo cells were treated with 20 µM THC (Sigma, Cat. No. T4764) for 48 hr. To induce syncytialization, BeWo cells were treated with epidermal growth factor (EGF; 50 ng/ml) (Johnstone, Sibley, Lowen, & Guilbert, 2005) for 48 hr to facilitate monolayer formation and subsequently differentiated using forskolin (FSK; 50 µM) (Orendi, Gauster, Moser, Meiri, & Huppertz, 2010), along with the replenishment of EGF, to promote fusion for an additional 48 hr. The media were supplemented with 20 µM THC concomitant with the addition of FSK.

### 2.2 Assessment of cellular viability

The reduction of 3‐(4,5‐dimethylthiazol‐2‐yl)‐5‐(3‐carboxymethoxyphenyl)‐2‐(4‐sulfophenyl)‐2H‐tetrazolium (MTS assay) to a formazan salt was used as a measure of cell viability. BeWo cells were subcultured into a 96‐well plate at a density of 1 × 10^4^ cells/well. Control wells containing media without cells were allocated to determine background absorbance. Cells were treated with THC for 48 hr. BeWo cells were treated with 20 µl of CellTiter 96® AQueous Non‐Radioactive Cell Proliferation Assay (Promega Corp., Cat. No. G5421) for 2 hr at 37°C in a humidified, 5% CO_2_ atmosphere. The absorbance was immediately recorded at 490 nm using a 96‐well plate reader (Miltiskan® Spectrum spectrophotometer; Thermo Scientific, Canada). The results are presented as percent of the MTS absorbance obtained in untreated cells (100%).

### 2.3 Assessment of plasma membrane integrity

As a measure of plasma membrane integrity, lactate dehydrogenase (LDH) release into culture supernatants was detected spectrophotometrically at 490 and 680 nm, using the Pierce LDH Cytotoxicity Assay Kit (cat. no. 88953), according to the manufacturer's recommended protocol. The results are presented as fold increase in the absorbance measured (normalized to untreated cells).

### 2.4 Immunofluorescence

BeWo cells were seeded on coverslips (1 × 10^5^ cells/cm^2^) and treated with THC or vehicle. Cells were immunostained according to previously established procedures (Wong et al., 2018). Briefly, the primary antibody against E‐cadherin (Abcam, UK) was diluted (1:500) in phosphate buffered saline with Tween (PBS‐T) with 0.1% bovine serum albumin (BSA) and incubated with the fixed cells overnight at 4°C. Cells were washed twice with PBS‐T. A goat anti‐rabbit antibody conjugated to AlexaFluor 488 was diluted (1:100) in PBS‐T with 0.1% BSA. Cells were incubated with the secondary antibody solution for 2 hr at room temperature in the dark. After washing twice with PBS‐T, cells were incubated with 4′,6‐diamidino‐2‐phenylindole (DAPI, 1.5 µg/ml, diluted in PBS‐T and 0.1% BSA) for 5 min in the dark. Cells were washed twice with PBS‐T and mounted onto microscope slides with Fluoromount™ (Diagnostic Biosystems Inc., USA). Coverslips were imaged with a Nikon Eclipse Ti‐E (Nikon Instruments Inc., USA). Five fields of view were captured per sample. An average fusion percentage from the various fields of view was calculated and was used to quantify cell fusion. Total cell fusion percentage was calculated ((total number of nuclei in fused cells/total number of nuclei) × 100%). Fused cells were counted as cells that had two or more nuclei per continuous membrane as visualized by E‐cadherin staining. Counts were performed by two individuals blinded to the treatment groups.

### 2.5 Enzyme‐linked immunosorbent assay

BeWo cells were seeded (1 × 10^5^ cells/cm^2^) on 96‐well plates and treated with corresponding culture conditions. Supernatants were collected and the levels of secreted hCGβ protein were analyzed by enzyme‐linked immunosorbent assay as previously described by our laboratory (Wong et al., 2018). The concentration of hCGβ was normalized to total cell lysate in each well.

### 2.6 DCFDA assay (2′,7′‐dichlorofluorescin diacetate)

BeWo cells were seeded in a black, clear bottom 96‐well microplate at a cell density of 3 × 10^4^ cells/well; differentiated and treated with THC as described above. ROS was detected using the Abcam DCFDA Cellular ROS Detection Assay Kit (cat. no. ab113851) as per the manufacturer's recommended protocol. The resulting fluorescent signal was quantified (BioTek Synergy 4) at excitation and emission wavelengths of 485 and 535 nm, respectively. Data were standardized as a percent of control after background (blank wells with media only) subtraction, followed by normalization to total protein content via the bicinchoninic assay (BCA) (Masaki, Izutsu, Yahagi, & Okano, 2009) which enables the standardization of ROS production as a function of cell number.

### 2.7 BCA assay

Protein concentration was determined by using the BCA (ThermoFisher Scientific, cat. no. 23252) with BSA (0–2,000 µg/ml) as a concentration standard. Total protein concentration was measured by quantifying the absorbance at 562 nm using a multiwell plate reader (Miltiskan® Spectrum spectrophotometer; Thermo Scientific, Canada).

### 2.8 RNA extraction and RT‐PCR

Cells grown on 12‐well plates were lysed with 500 µl of ice‐cold TRIzol™ reagent (Thermo Fisher Scientific, Canada). Total RNA isolation and quantification of gene expression (RT‐PCR) was performed as previously described by our research group (Wong et al., 2018). All genes and their respective primer sequences are listed in Table 1. Fold‐change mRNA expression was quantified using the double‐delta Ct analysis, normalized to housekeeping gene, 18S, then expressed as the relative fold change to the vehicle control sample expression.

**TABLE 1:** Primer sequences of human genes analyzed via RT‐PCR

Gene	Forward (5′→3′)	Reverse (5′→3′)
*18S*	CACGCCACAAGATCCCA	AAGTGACGCAGCCCTCTATG
*CGA*	GCAGGATTGCCCAGAATGC	TCTTGGACCTTAGTGGAGTGG
*CGB*	ACCCCTTGACCTGTGAT	CTTTATTGTGGGAGGATCGG
*DRP1*	AAACTTCGGAGCTATGCGGT	AGGTTCGCCCAAAAGTCTCA
*ERVFRD‐1*	GCCTACCGCCATCCTGATTT	GCTGTCCCTGGTGTTTCAGT
*ERVW‐1*	GTTAATGACATCAAAGGCACCC	CCCCATCTCAACAGGAAAACC
*GCM1*	CCTCTGAAGCTCATCCCTTGC	ATCATGCTCTCCCTTTGACTGG
*HSP60*	GAAGGCATGAAGTTTGATCG	TTCAAGAGCAGGTACAATGG
*HSP70*	GGAGTTCAAGAGAAAACACAAG	AAGTCGATGCCCTCAAAC
*IGF2*	GCCAATGGGGAAGTCGATGCTGG	GAGGCTGCAGGATGGTGGCG
*MFN1*	TTGGAGCGGAGACTTAGCAT	GCCTTCTTAGCCAGCACAAAG
*MFN2*	CACAAGGTGAGTGAGCGTCT	ACCAGGAAGCTGGTACAACG
*OPA1*	GCTCTGCATACATCTGAAGAACA	AGAGGCTGGACAAAAGACGTT
*PL*	GCCATTGACACCTACCAG	GATTTCTGTTGCGTTTCCTC
*SOD1*	AAAGATGGTGTGGCCGATGT	CAAGCCAAACGACTTCCAGC
*SOD2*	GCTCCGGTTTTGGGGTATCT	GATCTGCGCGTTGATGTGAG

### 2.9 Mitochondrial respiration assay

The mitochondrial oxygen consumption rate (OCR) was measured at 37°C in an XFe24 Extracellular Flux Analyzer (Agilent, Santa Clara, CA, USA). In brief, BeWo cells were plated at a density of 5 × 10^4^ cells/well in 250 µl culture media, in 24‐well microtiter plates. Syncytiotrophoblasts were differentiated and treated with THC as described above. After 48 hr, culture media were removed and replaced with XF base medium (Seahorse Bioscience, cat. no. 102365‐100) supplemented with 100 mM sodium pyruvate (ThermoFisher Scientific, cat. no. 11360‐070), 200 mM l‐glutamine (ThermoFisher Scientific, cat. no. 25030081), and 5 ml of 45% glucose solution (Millipore‐Sigma, cat. no. G8769), warmed to 37°C (pH 7.4). The assay medium was preequilibrated at 37°C for 1 hr. OCR was detected under basal conditions followed by the sequential injection of oligomycin (ATP‐synthase inhibitor), carbonyl cyanide‐4‐(trifluoromethoxy)phenylhydrazone (FCCP; mitochondrial respiration uncoupler), and rotenone combined with antimycin (electron transport blockers). These agents were injected through ports of the Seahorse Flux Pak cartridges to reach final concentrations of 1, 2, and 0.5 μM, respectively. The OCR value measured after oligomycin treatment represents the amount of oxygen consumption linked to ATP production, and that after FCCP injection denotes the maximal mitochondrial respiratory capacity of the cells. The final injection of rotenone and antimycin inhibits the flux of electrons through complexes I and III, respectively, and thus no oxygen is further consumed at complex V. The OCR reading after this treatment is primarily nonmitochondrial. OCR measurements were obtained using the Seahorse XFe24 Analyzer and the OCR values were normalized to the amount of protein content from each well. The detection of OCR was performed with four biological replicates per experiment, for each treatment condition, and repeated three more times.

### 2.10 Mitochondrial membrane potential

THC‐stimulated changes in the mitochondrial membrane potential (ΔΨm) were assessed using the fluorescent reagent tetraethylbenzimidazolylcarbocyanine iodide (JC‐1) with the JC‐1‐Mitochondrial Membrane Potential Assay kit (Abcam, cat. no. ab113850) following the manufacturer's protocol. BeWo cells were seeded at a density of 5 × 10^4^ cells/well and allowed to adhere overnight in a black, clear‐bottom 96‐well plate. Cells were treated with 20 µM THC for 48 hr. Following treatment, cells were washed once with 1X dilution buffer and then incubated with 20 µM JC‐1 dye in 1X dilution buffer for 10 min at 37°C, protected from light. JC‐1 dye was then removed, cells were washed once with 1X dilution buffer, and 100 µl of fresh 1X dilution buffer was added to each well. The red fluorescence in excitation (535 nm)/emission (590 nm) and green fluorescence excitation/emission (475 nm/530 nm) was measured using a Spark multimode microplate reader (Tecan Group Ltd.). Background fluorescence was subtracted from the fluorescence of treated cells, and then the ratio of red (polarized) fluorescence divided by that of green (depolarized) fluorescence was obtained.

### 2.11 CB1 and CB2 receptor antagonist treatments

To attenuate the effects of THC at the canonical cannabinoid receptors CB1 and CB2, BeWo cells were preincubated with selective CB1 antagonist AM281(1 µM) (Ford, Honan, White, & Hiley, 2002; Lan et al., 1999) and selective CB2 antagonist AM630 (1 µM) (Ford et al., 2002; Pertwee et al., 1995, 2010) for 30 min before the administration of THC (20 µM). This concentration of AM281 was previously shown to completely block the loss in mitochondrial membrane polarity in BeWo cells induced by WINN‐55,212, a synthetic cannabinoid, whereas AM630 attenuated this effect (Almada et al., 2017). Furthermore, this concentration of AM630 was previously demonstrated to reverse the THC‐induced inhibition of hCG secretion in BeWo cells (Costa et al., 2015).

### 2.12 Statistical analyses

All experiments were performed in biological triplicates (unless otherwise stated). Comparisons between two groups were performed using Student's *t* test. One‐ or two‐way analysis of variance and Bonferroni post hoc tests were used to compare datasets with more than two groups. Data are reported as means ± *SEM*. *p* < .05 was considered significant. The experimental parameters were analyzed using XFe Wave software V2.6.1 and Excel (for OCR measurements) and GraphPad Prism software V6.0.

## 3 RESULTS

### 3.1 THC reduces proliferation as assessed by MTS assay and inhibits syncytialization in trophoblast cells

The MTS assay was used to quantify change in the number of cells and the LDH assay was used to determine cytotoxicity in response to THC treatment. Syncytiotrophoblasts treated for 48 hr with a range of THC concentrations (Fig. S1b) demonstrated decreased proliferation between 20 and 30 µM (Fig. S1b, *p* < .01). Proliferation of the cytotrophoblast population was not significantly affected by the range of THC used in our study (Fig. S1a). The LDH assay demonstrated that no cell death was evident over the concentration range of THC tested in either cellular subpopulation (Fig. S1c,d).

**FIGURE 1:**
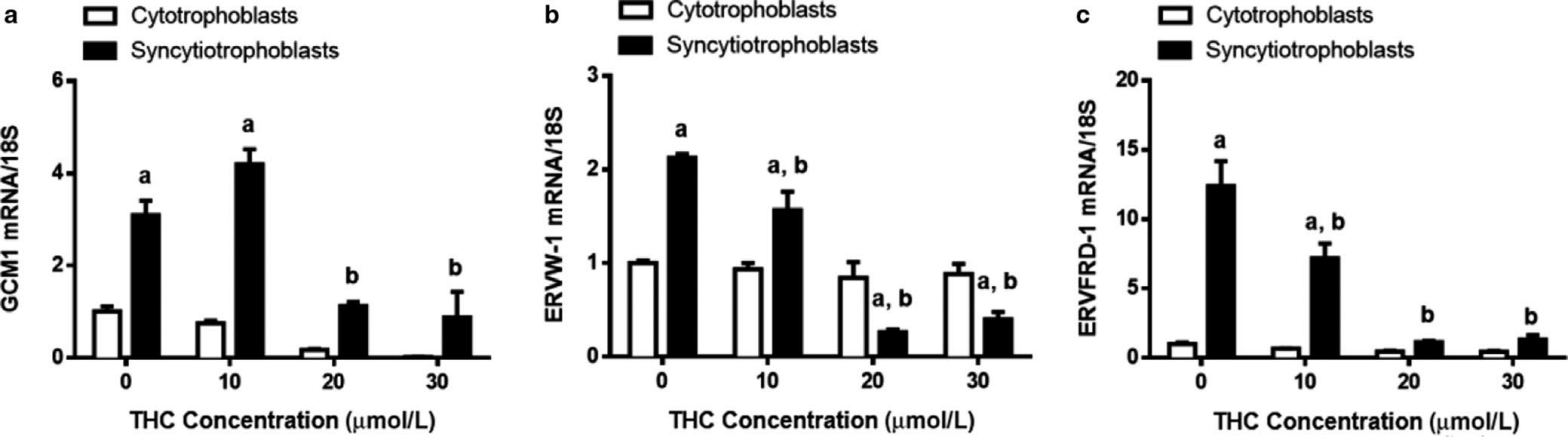
Transcriptional markers of syncytialization and biochemical differentiation are significantly suppressed by THC. Summary histograms of relative *GCM1* (a), *ERVW‐1* (b), and *ERVFRD‐1* (c) transcript expression in each treatment group normalized to 18S, then compared to the gene in the vehicle control. Significant differences were determined by a two‐way ANOVA, followed by a Bonferroni post hoc test. Data are presented as means ± *SEM* (*n* = 3). Different letters denote significant differences compared to the cytotrophoblast (a) or syncytiotrophoblasts (b) vehicle controls. *p* < .05 (a); *p* < .01 (b); *p* < .001 (c)

**FIGURE 2:**
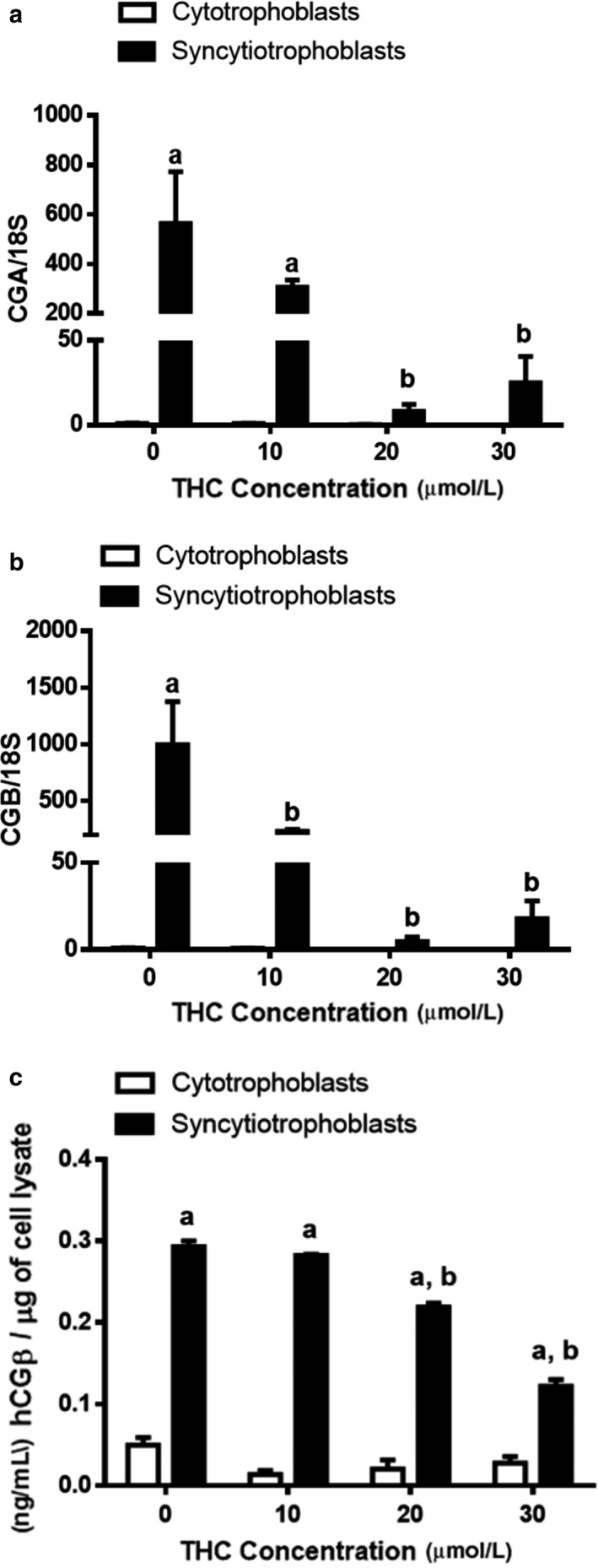
mRNA expression of CG subunits and secretion of hCGβ is significantly decreased by THC exposure. Summary histograms of relative *CGA* (a) and *CGB* (b) are shown. (c) Media were collected 48 hr after the administration of THC. The concentration of hCGβ was normalized to total cell lysate in each well. Significant differences were determined by a two‐way ANOVA, followed by a Bonferroni post hoc test. Data are presented as means ± *SEM* (*n* = 3). Different letters denote significant differences compared to the cytotrophoblast (a) or syncytiotrophoblast (b) vehicle controls. *p* < .01 (a, b); *p* < .0001 (c)

**FIGURE 3:**
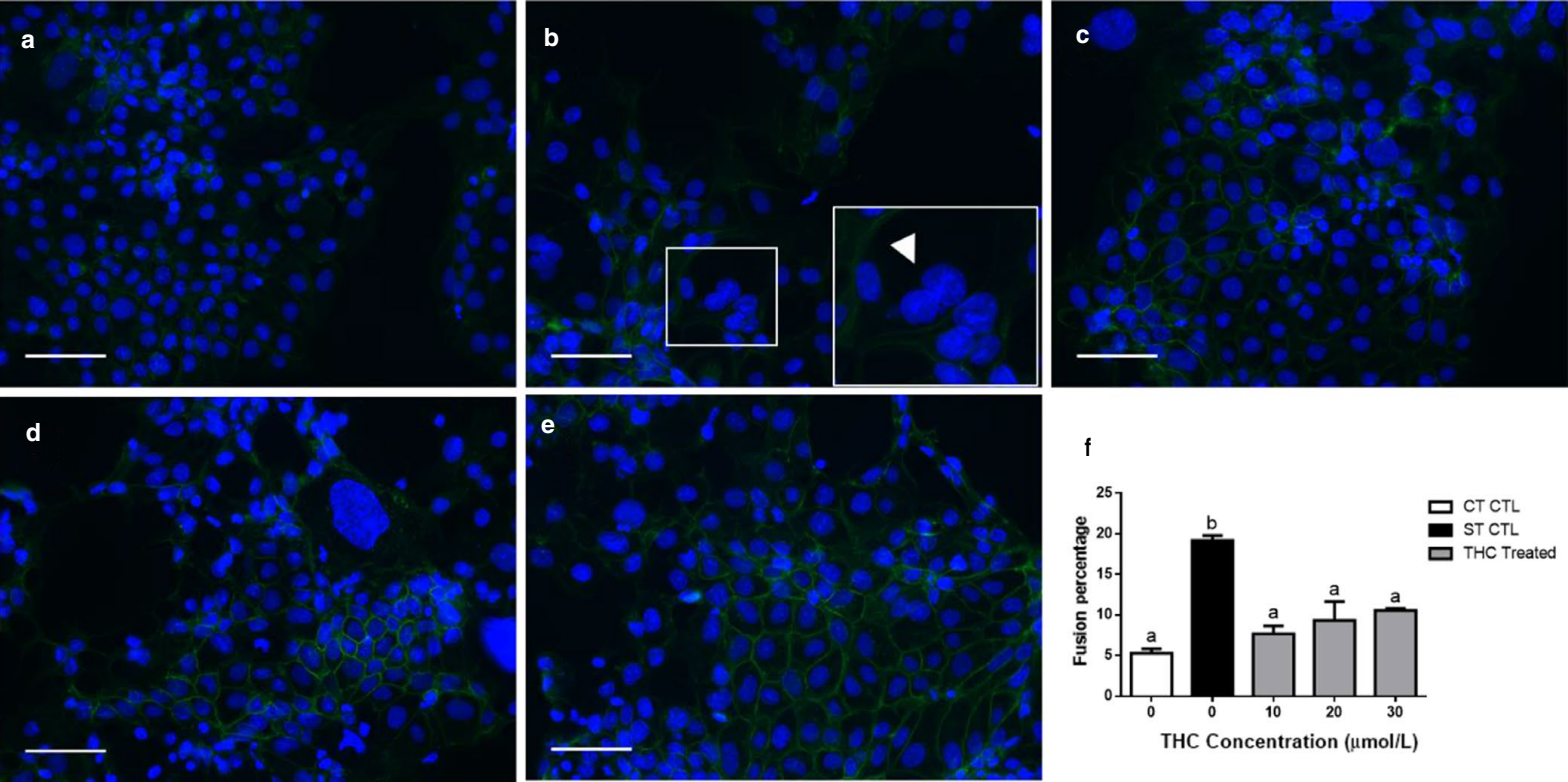
BeWo cell fusion is reduced following exposure to THC. BeWo cells showing immunofluorescent staining (a–d) for E‐cadherin distribution at the cell membrane using FITC‐conjugated secondary antibody (green) and counterstained with DAPI identifying the nuclei (blue). Representative fluorescent microscopy images (magnification 200×) are shown (a–e), selected from five random, nonoverlapping regions per treatment group (*n* = 3); scale bars indicate 100 µm. a = CT control; b = ST control (EGF, FSK); c = 10 µM THC, EGF, FSK; d = 20 µM THC, EGF, FSK; e = 30 µM THC, EGF, FSK. Insets represent close‐up magnifications of E‐cadherin localization with an example of multinucleated syncytiotrophoblasts (arrowhead in panel b). Cell counts were performed by two researchers and the average was used to determine the fusion percentage across all groups (f). Significant differences were determined by a one‐way ANOVA, followed by a Bonferroni post hoc test. Data are presented as means ± *SEM* (*n* = 3). Different letters denote significant differences among control and treated groups at *p* < .01

To obtain cytotrophoblast and syncytiotrophoblast populations, BeWo cells were cultured in the absence of (cytotrophoblast), or presence of EGF + FSK (syncytiotrophoblast), to induce differentiation. We evaluated the impact of THC on the cytotrophoblast population as well as on the fusion of cytotrophoblasts to form a syncytium. At the transcript and protein levels, various markers of syncytialization and biochemical differentiation were significantly reduced in response to THC treatment (Figures 1 and 2). As expected, the indices for the above processes did not change significantly within the cytotrophoblast population. In the syncytiotrophoblast population, we observed a marked increase in the expression of transcripts‐associated BeWo fusion in comparison to undifferentiated cells. This increase was reduced by four‐ to sevenfold for glial cell missing 1 (*GCM1*, Figure 1a, *p* < .05), endogenous retrovirus group W member 1 (*ERVW‐1*, Figure 1b, *p* < .01), and a more than 10‐fold reduction for endogenous retrovirus group FRD member 1 (*ERVFRD‐1*, Figure 1c, *p* < .001) following THC exposure. We also observed more than a 50‐fold reduction in the transcripts for hCG α and *β* (*CGA* and *CGB*, Figure 2a,b, *p* < .01); this corresponded to a twofold reduction (Figure 2c, *p* < .0001) in the release of hCG *β* over the concentration range of THC tested. To compliment these findings, we assessed cellular fusion using immunofluorescent staining. The presence of two or more nuclei within a cell boundary, stained using E‐cadherin, was defined as syncytialization. Treatment with THC over a 48‐hr time course increased the number of nuclei surrounded by E‐cadherin–positive boundaries. This translates to a decrease in fusion percentage (total number of nuclei in fused cells/total number of nuclei) × 100%) (Figure 3, histogram in panel F).

### 3.2 PL and IGF2 are markedly reduced following THC treatment

**FIGURE 4:**
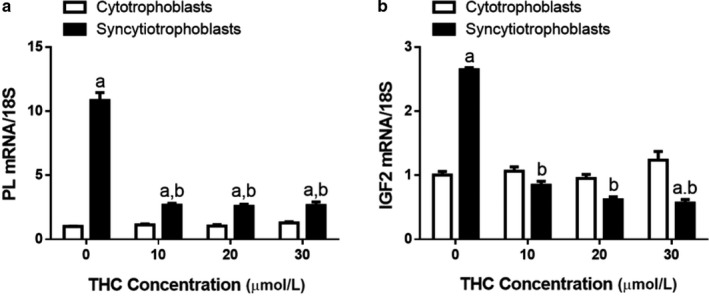
Expression of insulin‐like growth factor 2 (IGF2) and PL in differentiated BeWo cells is reduced following THC treatment. Panel a: *PL* transcript. Panel b: *IGF2* transcript. Data are presented as mean ± *SEM*, *n* = 3. Different letters denote significant differences compared to the cytotrophoblast (a) or syncytiotrophoblast (b) vehicle controls. *p* < .0001 (a); *p* < .01 (b)

Following 48 hr of THC treatment, the expression of *PL* and insulin‐like growth factor 2 (*IGF2*) transcripts were reduced by 5‐ (Figure 4a, *p* < .0001) and 2.5‐fold (Figure 4b, *p* < .01), respectively.

### 3.3 THC affects markers of mitochondrial fragmentation in trophoblast cells

**FIGURE 5:**
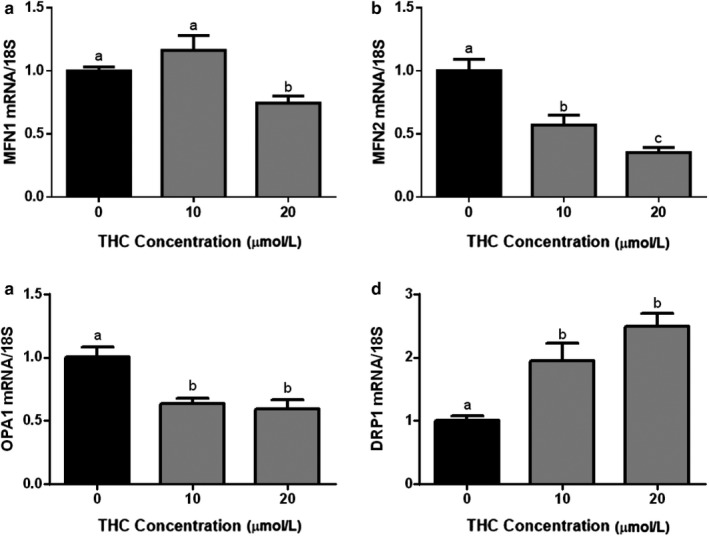
Mitochondrial fusion and fission transcripts in differentiated BeWo cells are altered following exposure to THC. Summary histograms shown of relative *MFN1* (a), *MFN2* (b), *OPA1* (c), and *DRP1* (d) transcript expression in each treatment group were normalized to 18S, and then compared to the gene in the vehicle control group. Significant differences were determined by a one‐way ANOVA, followed by a Bonferroni post hoc test. Data are presented as means ± *SEM* (*n* = 3). Different letters denote significant differences among control and treated groups at *p* < .01 (a); *p* < .001 (b–d)

To explore the mechanisms by which THC could influence mitochondrial fragmentation, we examined the transcript abundance of some key factors that regulate mitochondrial morphology/dynamics in differentiated BeWo cells. We analyzed the change in the expression of the mitochondrial fission mediator dynamin‐related protein 1 (*DRP1*), and fusion mediators mitofusin 1 and 2 (*MFN1*, *MFN2*) and optic atrophy 1 (*OPA1*). BeWo cells treated for 48 hr with THC exhibited a significant reduction in the expression of the fusion effectors (Figure 5a, *p* < .01, Figure 5b,c *p* < .001). This was concomitant with a 100% increase in the expression of *DRP1*, the fission effector (Figure 5d, *p* < .001).

### 3.4 Intracellular stress responses and defenses are increased upon THC treatment in BeWo cells, concomitant with an increase in oxidative stress

**FIGURE 6:**
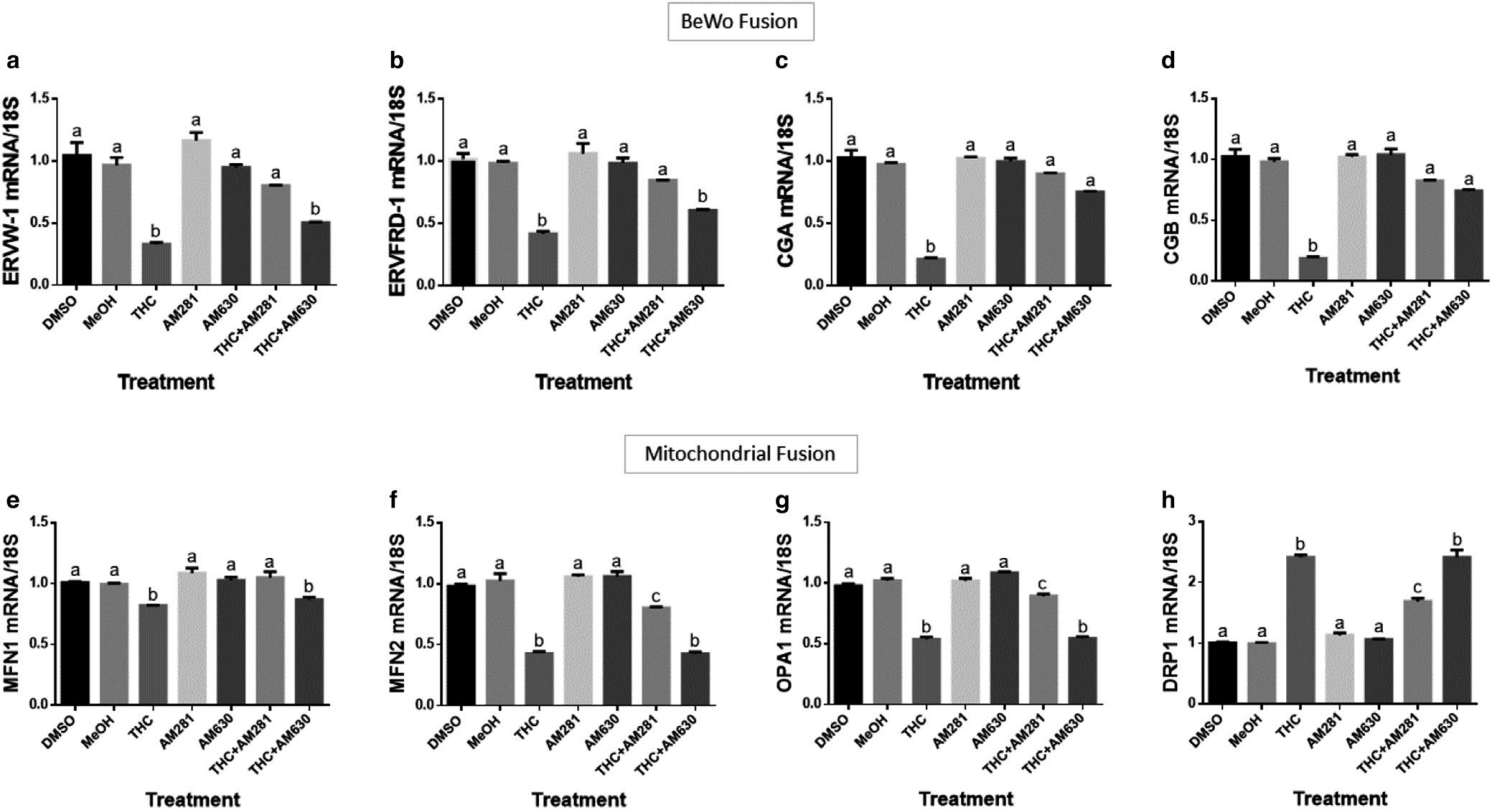
THC‐CB receptor binding reduces markers of syncytialization and mitochondrial fusion. Summary histograms are shown of relative *ERVW‐1* (a), *ERVFRD‐1* (b), *CGA* (c), *CGB* (d), *MFN1* (e), *MFN2* (f), *OPA1* (g), and *DRP1* (h) transcript expression in each treatment group as indicated. Significant differences were determined by a one‐way ANOVA, followed by a Bonferroni post hoc test. Data are presented as means ± *SEM* (*n* = 3). Different letters denote significant differences among vehicle control (1% v/v) and treated groups at *p* < .0001 (a–h). THC, 20 µM; AM281, 1 µM; AM630, 1 µM

THC exposure resulted in a twofold increase in intracellular reactive oxygen species (ROS) generation (Figure 6e, *p* < .05). Increased levels of intracellular ROS have been shown to induce the transactivation of heat‐shock factor 1 (*HSF1*) (Lee et al., 2015), the principle transcription factor of heat‐shock protein 60 and 70 (*HSP60* (Ciocca, Arrigo, & Calderwood, 2013) and *HSP70* (Ciocca et al., 2013; Lee et al., 2015)). Following 48 hr of THC treatment in BeWo cells, we observed a 5‐ and 2.5‐fold upregulation of *HSP60* and *HSP70* transcripts, respectively (Figure 6a,b, *p* < .0001). Furthermore, transcript levels of manganese superoxide dismutase (*SOD2*; the mitochondrial isoform) and copper zinc superoxide dismutase (*SOD1*; the cytosolic isoform) (Figure 6c, *p* < .0001, Figure 6d, *p* < .001); two enzymes routinely associated with the regulation of cellular oxidative stress (Thompson & Al‐Hasan, 2012) were also upregulated in response to THC exposure.

### 3.5 CB1 or CB2 receptor antagonists attenuated THC‐induced inhibition of BeWo cell syncytialization and mitochondrial fusion

To determine whether THC mediated the observed impairments to BeWo cell fusion or mitochondrial fusion through CB1 and/or ‐CB2, BeWo cells were pretreated with AM281 (CB1 antagonist, 1 µM) and AM630 (CB2 antagonist, 1 µM) for 30 min followed by treatment with THC at 20 µM for 48 hr. THC significantly reduced all the transcriptional markers of syncytial formation (*ERVW‐1, ERVFRD‐1, CGA,* and *CGB*, Figure 6a–d) and mitochondrial fusion (*MFN1. MFN2, and OPA1*, Figure 6e–g) while concomitantly increasing the transcriptional expression of *DRP1* (Figure 6h), a marker of mitochondrial fission. CB1 antagonism completely abolished the effects on *ERVW‐1, ERVFRD‐1, CGA,* and *CGB*. However, the effects on *MFN2*, *OPA1,* and *DRP1* (Figure 6f–h) expression were only partially attenuated. The THC‐induced reduction on *CGA* and *CGB* transcripts was completely blocked in the presence of the CB2 antagonist (Figure 6c,d) while the remaining transcripts remained unchanged.

### 3.6 THC alters mitochondrial membrane potential

**FIGURE 7:**
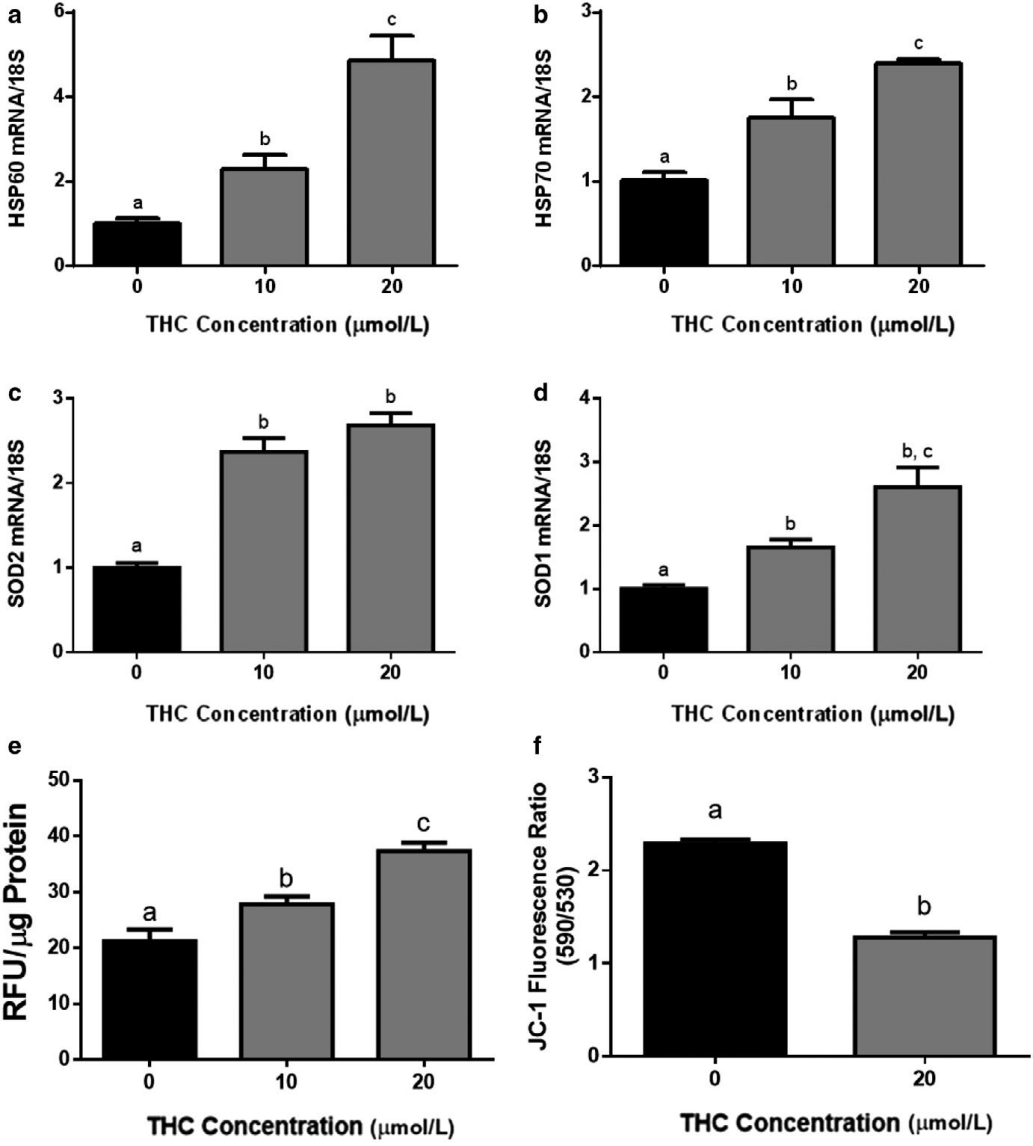
Stress responses, intracellular defenses, ROS production, and mitochondrial membrane potential are altered in ST cells upon THC treatment. Summary histograms are shown of relative *HSP60* (a), *HSP70* (b), *SOD2* (c), and *SOD1* (d) transcript expression. Each treatment group was normalized to 18S, and then compared to the gene in the vehicle control group. (e) DCFDA assays were performed to determine intracellular ROS levels following THC treatment. Tert‐butyl hydrogen peroxide (TBHP) solution (100 µM) was used as the positive control. Results were normalized to the protein content of cell lysates. Significant differences were determined by a one‐way ANOVA, followed by a Bonferroni post hoc test. Data are presented as means ± *SEM* (*n* = 3). Different letters denote significant differences among control and treated groups at *p* < .0001 (a–c); *p* < .001 (d); *p* < .05 (e). RFU = relative fluorescence units. (f) Mitochondrial depolarization in response to THC exposure. A Student's *t* test indicates significance (*p* < .001) between vehicle‐treated and THC‐treated samples

We used JC‐1, a selective ΔΨm dye, to explore the role of mitochondrial dysfunction in THC‐induced responses. Because JC‐1 fluorescence shifts from red to green with membrane depolarization, changes in ΔΨm were quantified by changes in the JC‐1 red/green fluorescence intensity ratio. Treatment with 20 µM THC for 48 hr significantly decreased the JC‐1 red/green fluorescence intensity ratio by 44.1% in syncytiotrophoblasts, compared to untreated controls (Figure 7f, *p* < .001, respectively).

### 3.7 THC reduces oxygen consumption rate and ATP production in differentiated BeWo cells

**FIGURE 8:**
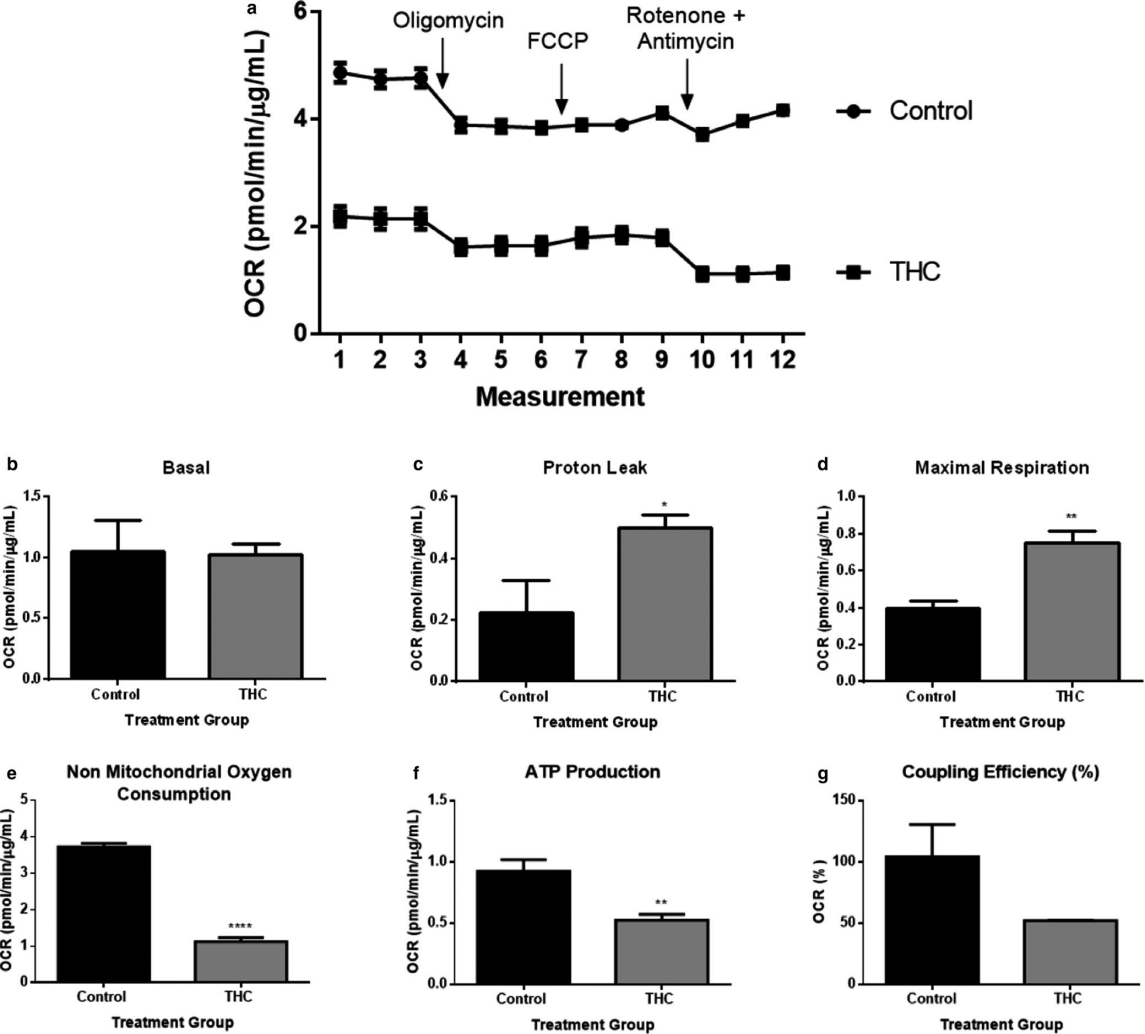
Mitochondrial function in ST cells is impaired following 20 µM THC treatment. OCR was detected under basal conditions followed by the sequential injection of oligomycin (ATP‐synthase inhibitor), carbonyl cyanide‐4‐(trifluoromethoxy)phenylhydrazone (FCCP; mitochondrial respiration uncoupler), and rotenone combined with antimycin (electron transport blockers). These agents were injected through ports of the Seahorse Flux Pak cartridges to reach final concentrations of 1, 2, and 0.5 μM, respectively. The OCR value measured after oligomycin represents the amount of oxygen consumption linked to ATP production, and that after FCCP injection denotes the maximal mitochondrial respiratory capacity of the cells. The final injection of rotenone/antimycin inhibits the flux of electrons through complexes I & III, and thus no oxygen is further consumed at complex V. The OCR reading after this treatment is primarily nonmitochondrial and is due to cytosolic oxidase enzymes. OCR measurements were obtained using the Seahorse XFe24 Analyzer and the OCR values were normalized to the amount of protein content from each well using the BCA assay (μg/ml). The detection of OCR was performed with four biological replicates per experiment, for each treatment condition, and repeated three more times (see Figure S2 for experiments 2–4). Group mean and *SEM* (a–g) are displayed. Arrows indicate addition of the respective compounds. Significance was assessed by Student's *t* test (*p < .05, **p < .01, ****p < .0001). (a) Representative oxygen consumption rate (OCR) tracing, (b) basal respiration, (c) proton leak, (d) maximal respiration, (e) nonmitochondrial oxygen consumption, (f) ATP production, and (g) coupling efficiency (%)

To further investigate the changes in mitochondrial function that occurred in BeWo cells in response to THC, a Cell Mito Stress assay kit was used to detect the OCR. Trophoblast cells were treated with 20 µM THC for 48 hr before exposure to 1 µM oligomycin, 2 µM FCCP, and 0.5 µM rotenone and antimycin. As demonstrated in Figure 8a, THC reduced OCR in BeWo cells, suggesting that THC attenuates mitochondrial respiration. When compared to untreated cells, THC did not significantly affect basal respiration in syncytiotrophoblasts (Figure 8b). In syncytiotrophoblasts, THC significantly reduced nonmitochondrial respiration by 69.8% (Figure 8e, *p* < .0001) and ATP production by 43.2% (Figure 8f, *p* < .01), while markedly increasing proton leak by 122.2% (Figure 8c, *p* < .05). Interestingly, maximal respiration was significantly increased by 87.5% (*p* < .01) in the syncytiotrophoblasts upon THC treatment (Figure 8d).

## 4 DISCUSSION

The placenta is an autonomous and transient organ that is critical for the maintenance of pregnancy and materno‐fetal exchanges. Aberrant formation and function of the placenta is associated with pregnancy disorders such as preeclampsia and intrauterine growth restriction (Gupta et al., 2015). The syncytium is critical in forming the materno‐fetal interface. In vivo, formation of the syncytium is dependent upon the differentiation and fusion of the underlying stem cells, the cytotrophoblasts. Cytotrophoblast differentiation is characterized by biochemical and morphological changes, including upregulation and expression of hPL, hCGβ, and production of estrogen and progesterone (Gude et al., 2004; Gupta et al., 2015; Wice, Menton, Geuze, & Schwartz, 1990). As the placenta is an organ with high endocrine activity, hormone production and release are vital aspects of a placental/trophoblast model. hCG is a secreted peptide hormone that mediates important physiological events such as progesterone production (Weedon‐Fekjær & Taskén, 2012), placental vascularization (Cole, 2010), immune protection for mother and fetus (Wan et al., 2007), fetal organogenesis (Cole, 2010), and promotes the continuous differentiation of cytotrophoblasts to syncytiotrophoblasts throughout gestation (Cole, 2010; Pidoux et al., 2007; Shi, Lei, Rao, & Lin, 1992).

To evaluate whether THC treatment impacted these critical trophoblast functions, we used the BeWo cells as a model to study proliferation, differentiation, and syncytialization, as they are the only cell line which can be induced to differentiate and syncytialize in a protein kinase A‐dependent process (Knerr et al., 2005); emulating what is observed in trophoblasts in vivo, in humans (Knerr et al., 2005; Pidoux, 2015). While there are acknowledged differences between isolated human primary trophoblast cells and cultured trophoblast cell lines, the use of established cell lines have been advocated for by many researchers (Bode et al., 2008; Orendi et al., 2010; Rothbauer et al., 2017) and reviewed by Sullivan (2004).

In the present study, we assessed the effect of THC exposure on the process of BeWo fusion by quantifying (a) markers of syncytialization and indicators of trophoblast fusion; (b) hCG secretion; and (c) the observation of multinucleated syncytia via immunofluorescent staining. We demonstrate that exposure of BeWo cells to THC results in a significant reduction in *CG* gene transcription mediated by both CB1 and CB2 and attenuated the release of hCGβ from the STs. Furthermore, THC exposure also resulted in reductions in the transcriptional markers of syncytialization largely mediated via CB1 binding, and a reduction in the percentage of fused trophoblasts. Aberrant syncytialization has been implicated in preeclampsia (Roland et al., 2016), and several reports have indicated that the master players in syncytialization, including *GCM1* and the syncytins (Vargas et al., 2009), are also downregulated in preeclampsia (Langbein et al., 2008; Orendi et al., 2010; Roland et al., 2016). In this way, THC may interfere with the mechanisms involved in trophoblast fusion and, thus, be implicated in poor pregnancy outcomes.

One of the primary functions of the human placenta is to produce hormones and other mediators which are critical for pregnancy success. These include hPL and IGFs, molecules which are implicated in fetal growth and development (Constância et al., 2002; Forbes, Westwood, Baker, & Aplin, 2008; Mcintyre et al., 2000). Treatment of trophoblasts with THC for 48 hr led to reduced transcriptional expression of both markers, which further suggests perturbed syncytial function. Various studies demonstrate that infants that are born small for their gestational age (SGA) were exposed to decreased levels of hPL and IGFs when compared to infants of normal pregnancies (Koutsaki et al., 2011; Mcintyre et al., 2000; Mirlesse et al., 1993). These growth factors were measured in maternal serum, plasma, and term placental tissue, respectively. hPL is a polypeptide hormone mainly secreted by syncytiotrophoblasts and its overall function is that of regulation of fetal and maternal lipid and carbohydrate metabolism (Costa, 2016b). Furthermore, hPL and IGFs are also potent stimulators of tissue growth and regulate the metabolic status of both mother and fetus (Forbes et al., 2008). IGF1 and IGF2 are expressed by fetal tissues and placenta; however, reduced fetal growth in mice lacking the placental‐specific transcript of *IGF2* (Constância et al., 2002) is associated with reduced placental mass (Sibley et al., 2004).

Birth weight is indicative of future risk of metabolic disorders including obesity, diabetes, and cardiovascular disease (Freemark, 2010). Healthy fetal growth and weight gain is dependent upon maternal metabolism, and placental development and function (Anthony, Pratt, Liang, & Holland, 1995; Freemark, 2010). The possibility that THC‐mediated changes in the placenta can restrict fetal growth is supported by the recent finding of Natale et al. (2020). Using a rat model of pregnancy, Natale et al. (2020) reported symmetrical fetal growth restriction following in utero exposure to THC (Natale et al., 2020). Furthermore, this group also demonstrated an increase in the labyrinth region of the placenta, leading to an overall greater mass for placentae from dams exposed to THC. Changes in placental vascularization, resulting from the development of narrower vascular networks, has also been observed in women who smoked cannabis without the confounding coexposure of alcohol or tobacco (Chang et al., 2018). Thus, the effect of THC on the reduced expression of *hPL* and *IGF2* in our in vitro system may contribute to the better understanding of mechanisms linking exposure of the syncytium to THC and outcomes reported in SGA fetuses.

Oxidative stress and mitochondrial dysfunction in the placenta have been previously associated with SGA offspring (Mandò et al., 2014). In fact, the regulation of oxidative stress is a critical parameter for normal progression of pregnancy (Holland et al., 2017) and excessive placental oxidative stress is characteristic of several gestational disorders, such as intrauterine growth restriction and preeclampsia (Holland et al., 2017). Mitochondria are known to contribute to placental oxidative stress (Myatt & Cui, 2004) and have also been shown to be negatively impacted by THC (Costa et al., 2015). Recent work by Lojpur et al. (2019) suggested that 15–30 µM THC triggered reduced mitochondrial oxidative phosphorylation and increased endoplasmic reticulum (ER) stress in unfused BeWo cytotrophoblasts. However, mitochondrial function and signals play a wide range of roles in the placenta including the regulation of syncytialization (Maloyan et al., 2012). To advance the understanding of how THC may reduce mitochondrial function and increase syncytiotrophoblast stress, we investigated the consequences of THC exposure on syncytiotrophoblast mitochondrial respiratory function, dynamics, antioxidant enzyme expression, as well as cellular ROS production and markers of cellular stress.

The MTS and LDH assays measure different aspects of cellular function. The MTS assay quantifies mitochondrial metabolic activity and indirectly reflects cellular proliferation. Conversion of MTS to its purple formazan precipitate is mediated by mitochondrial dehydrogenases. This precipitate, measured spectrophotometrically upon solubilization, is directly proportional to the number of viable cells (Rai et al., 2018), while the LDH assay allows for the assessment of plasma membrane integrity (Moghimi et al., 2005). We demonstrate that at 20 µM THC, mitochondrial metabolic activity was impaired while leaving the plasma membrane unperturbed. Furthermore, we show that THC, partly mediated via canonical CB1, also perturbs mitochondrial dynamics in trophoblasts as indicated by the increase in the markers of fission, *DRP1*, and the decrease in the markers of fusion, *MFNs* and *OPA1*. Interestingly, antagonism of CB2 did not normalize the THC‐mediated reduction in *MFN2*, *OPA1,* and *DRP1*. A healthy mitochondrial reticulum is fundamental for ATP synthesis, regulation of apoptosis, calcium signaling, and ROS production (Poidatz, Dos Santos, Loı, et al., 2015). Healthy mitochondrial reticulum is also an important indicator of overall cellular health and changes in its morphology is indicative of cellular stress (Eisner, Picard, & Hajnóczky, 2018; Zemirli, Morel, & Molino, 2018). We therefore assessed common indicators of oxidative and cellular stress. We observed elevated levels of cellular ROS, increased expression of *HSP60* and *HSP70* as well as *SOD1* and *SOD2*. Normally, ROS generation by the mitochondria is tightly controlled by SOD2 in mitochondria and SOD1 in the cytosol (Wang & Walsh, 1998). In our study, the BeWo cells demonstrate the capacity to compensate for the THC‐induced increase in ROS generation by increasing the synthesis of *SOD2* and *SOD1* transcripts. Because intracellular ROS may indirectly regulate *DRP1* in physiological and pathological processes (Gan et al., 2014; Guo, Sesaki, & Qi, 2014), our results raise the question whether increased *DRP1* expression leads to trophoblast dysfunction driven by oxidative stress in our in vitro model.

Decreased mitochondrial membrane potential, a signal characteristic of dysfunctional mitochondria, can trigger mitophagy or mitochondrial recycling (Twig et al., 2008; Twig & Shirihai, 2011). This process represents the targeted degradation of mitochondria and a mechanism by which a cell can maintain a healthy mitochondrial pool. Fission precedes mitophagy as this ensures that defective mitochondria are small enough to be engulfed by autophagosomes for subsequent breakdown in the autolysosome (Osellame, Blacker, & Duchen, 2012). A number of factors can contribute to mitochondrial stress and mitophagy, such as increased ROS and loss of mitochondrial membrane potential (Twig et al., 2008). We also demonstrate that similar markers of mitochondrial stress are triggered by THC treatment of BeWo cells and associated with markers of mitophagy. While mitochondria are replenished via division of the existing mitochondrial pool, deficits in this process may shift the equilibrium to a state where the cell contains defective mitochondria, perpetuating insufficient respiratory activity, and oxidative stress/damage (Osellame et al., 2012; Twig et al., 2008). Twig et al. (2008) demonstrated that mitochondria undergoing fission are more likely to undergo mitophagy and that this pool of mitochondria has a significantly lower mitochondrial membrane potential than the cellular pool of mitochondria that is fusion competent.

Critical for the maintenance of the mitochondrial membrane potential is the transfer of electrons through the ETC which results in the phosphorylation of ADP to form ATP via complex V–ATP synthase. Measurements of mitochondrial OCR in syncytiotrophoblasts allowed for direct quantification of the effect of THC on various aspects of the electron transport chain function. While we did not observe significant differences in basal respiration, ATP production was significantly reduced in THC‐treated cells. Interestingly, both the proton leak and maximal respiration demonstrated an increase following exposure to THC. Elevated proton leak may be a sign of mitochondrial inner membrane damage (Eisner et al., 2018) and may be connected to the decrease in membrane potential observed in THC‐treated cells. Furthermore, the reduced nonmitochondrial oxygen consumption observed in this study can be attributed to nonmitochondrial processes, such as enzymatic activity of oxygenases (Brand & Nicholls, 2011; Manfredi, Ang, Peker, Dagda, & Mcfarlane, 2019). Regarded as an indicator of bioenergetic health, nonmitochondrial OCR varies (Wagner, Venkataraman, & Buettner, 2011) and may decrease in the presence of stressors (Decleer et al., 2018) like ROS. It is well established that mitochondria are a target for the deleterious effects of reactive oxygen intermediate. Indeed, our group have demonstrated that 10 nM rotenone over 48 hr attenuated complex I activity in BeWo cells and led to increased ROS production concomitant with perturbed syncytialization (Walker et al., 2020).

Maximal respiratory rate is determined by several factors, including the functional capacity of the electron transport chain. The improved maximal respiration in the syncytiotrophoblasts concomitant with increased proton leak and decreased ATP production may be suggestive of a compensatory mechanism to preserve maximum oxygen consumption in the syncytiotrophoblasts. In fact, due to various perturbations, several mitochondrial compensatory responses have been reported in the literature (Haylett et al., 2016; Ireland, Maloyan, & Myatt, 2018; Manfredi et al., 2019) and references therein). These compensatory changes, along with the increase in *SOD1* and *SOD2* reported herein, may impart a protective effect on the placenta and the developing fetus in response to additional stressors, such as maternal obesity or preeclampsia. Lojpur et al. (2019) demonstrated that treatment of undifferentiated BeWo cells for 24 hr with 15 µM THC resulted in reduced state 3 respiration and this was associated with reduced expression of mitochondrial electron transport chain proteins. Lojpur et al. (2019) observed a reduction in basal respiration upon 24 hr of 15 µM THC exposure in undifferentiated BeWo cells (Lojpur et al., 2019), while we report no significant change in basal respiration in the differentiated BeWo cells. It may be important to consider that there have been reports of ultrastructural differences in the cristae structure of mitochondria from cytotrophoblasts and syncytiotrophoblasts (Castillo et al., 2011; Martinez, Kiriakidou, & Stauss, 1997). Syncytial mitochondria are thought to have lower ATP capacity and have smaller cristae structure to more effectively facilitate steroidogenesis (Castillo et al., 2011).

Mitochondrial dysfunction and the resulting oxidative stress are often associated with ER stress (Burton, Yung, & Murray, 2017). While we did not evaluate ER stress, it has been recently shown that in CTs, THC‐mediated reduction in mitochondrial function was associated with ER stress (Lojpur et al., 2019). There are numerous reports (Lojpur et al., 2019; Mizuuchi et al., 2016; Muralimanoharan et al., 2012; Natale et al., 2020; Shi et al., 2013; Walker et al., 2020) that have associated mitochondrial dysfunction and/or ER stress to adverse trophoblast function and birth outcomes. While we did not evaluate ER stress, it has been recently shown that in cytotrophoblasts, THC‐mediated reduction in mitochondrial function, as assessed by reduced OCR and impaired expression of ETC proteins, was associated with ER stress (Lojpur et al., 2019). Indeed, OXPHOS is, in part, regulated by crosstalk with intracellular calcium, such that efflux of calcium from the ER into the mitochondrial matrix signals an increase in energy demands. When mitochondrial calcium influx is excessive and prolonged, particularly when concomitant with oxidative stress, this may lead to various pathological changes, such as the release of apoptotic proteins from the matrix. Mitochondrial health is intimately linked to cellular viability and, when impaired, becomes associated with various disease states (Osellame et al., 2012).

Taken together, our findings demonstrate that THC attenuates the process of syncytialization and reduces the expression of hPL and IGF2, two important growth hormones connected to pregnancy success. Given the association between THC and reduced fetal growth, it is important to more clearly understand the mechanisms that underpin this association. Our report demonstrates that the observed mitochondrial dysfunction in syncytialized trophoblasts also contributes to increases in the markers of oxidative stress and changes to the dynamics of the mitochondrial reticulum. While it is important to verify these observations in primary trophoblasts, our data advance our understanding of the adverse effects of THC on the materno‐fetal interface.

## 5 COMPETING INTERESTS

The authors declare that they have no competing interests.

## AUTHORS’ CONTRIBUTIONS

OSW and SR designed the project. OSW, RR, HG, and ML performed the experiments. LLM performed the mitochondrial respiration assays. The manuscript was written by OSW and SR with editing by all authors.

## ETHICS APPROVAL AND CONSENT TO PARTICIPATE

Not applicable.

## CONSENT FOR PUBLICATION

Not applicable.

## Supporting information



Fig S1

Fig S2

## Data Availability

All data generated or analyzed during this study are included in this published article (and its supplementary information files).
