# Supplementary figures and images for: Delta‐9‐tetrahydrocannabinol disrupts mitochondrial function and attenuates syncytialization in human placental BeWo cells
Source: Physiol Rep. 2020 Jul 6;8(13):e14476. doi: 10.14814/phy2.14476 (PMC7336740; doi:10.14814/phy2.14476)

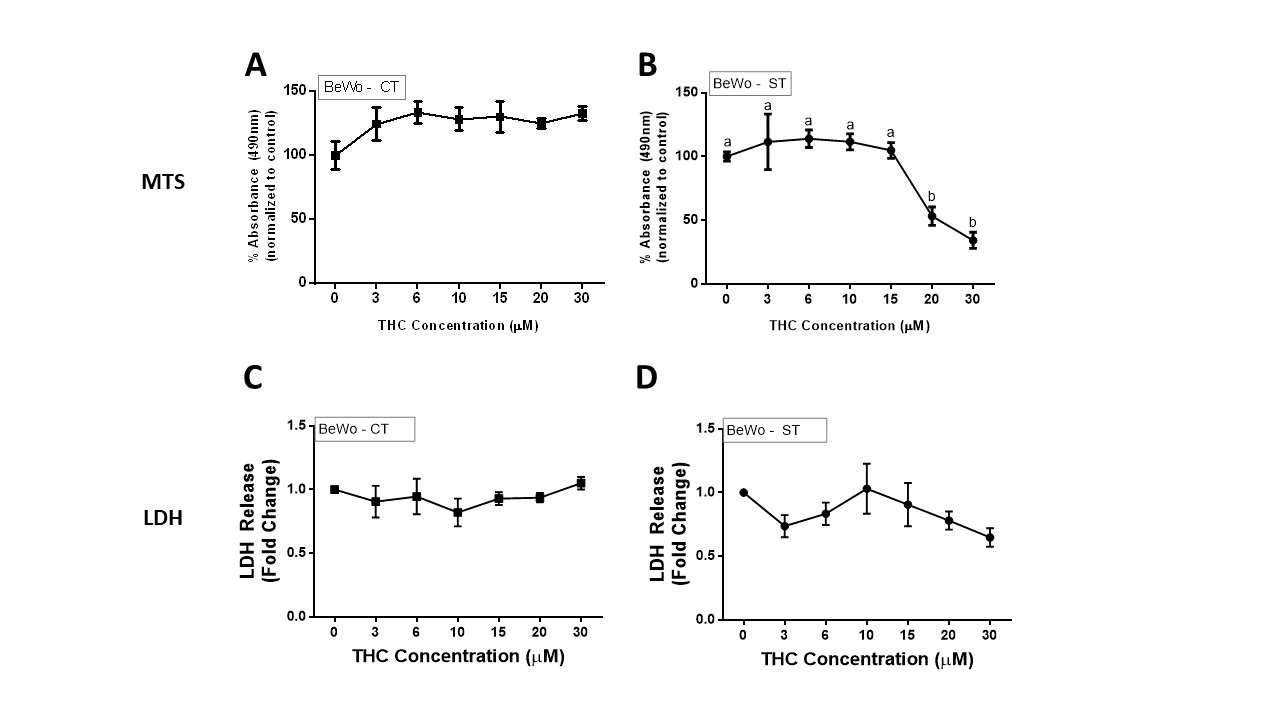

Supplement: Supplementary file 1 — Fig S1 [file PHY2-8-e14476-s001.png]

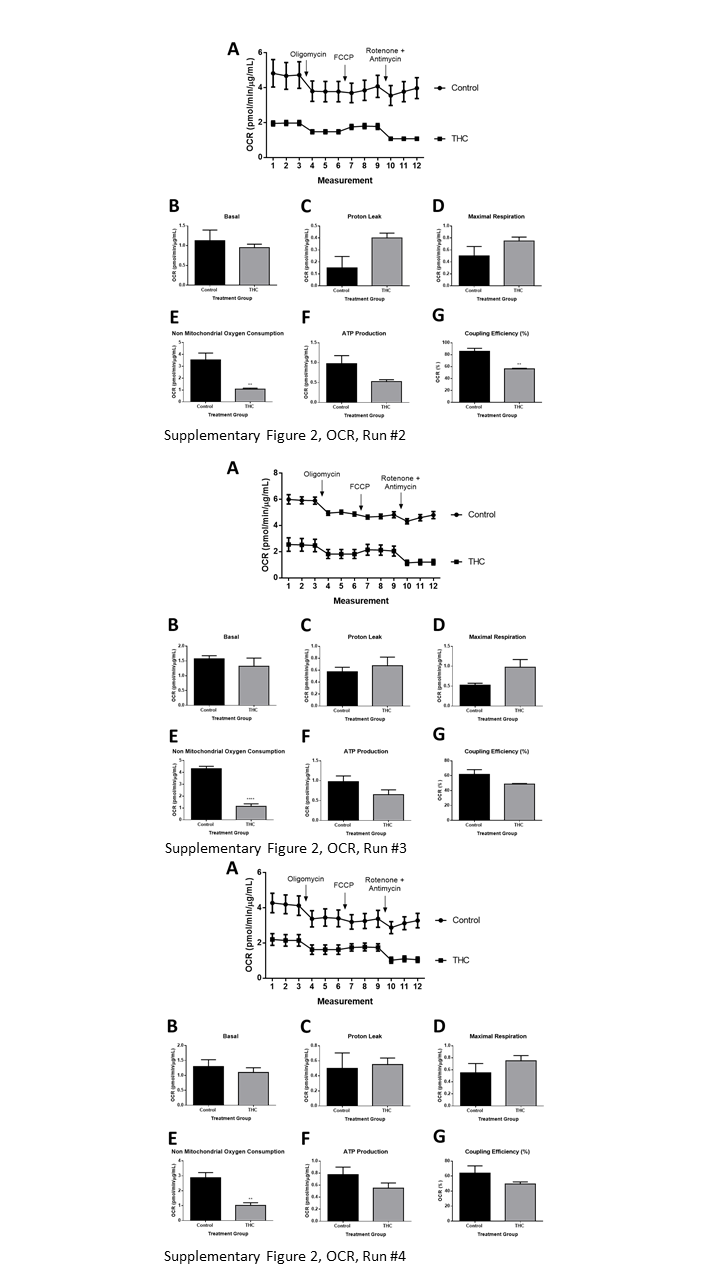

Supplement: Supplementary file 2 — Fig S2 [file PHY2-8-e14476-s002.png]
